# Cell-Intrinsic Roles for Autophagy in Modulating CD4 T Cell Functions

**DOI:** 10.3389/fimmu.2018.01023

**Published:** 2018-05-09

**Authors:** Elise Jacquin, Lionel Apetoh

**Affiliations:** ^1^INSERM, U1231, Dijon, France; ^2^Université de Bourgogne Franche-Comté, Dijon, France

**Keywords:** autophagy, T cell, CD4, differentiation, adaptive immunity, immunotherapy

## Abstract

The catabolic process of autophagy plays important functions in inflammatory and immune responses by modulating innate immunity and adaptive immunity. Over the last decade, a cell-intrinsic role for autophagy in modulating CD4 T cell functions and differentiation was revealed. After the initial observation of autophagosomes in effector CD4 T cells, further work has shown that not only autophagy levels are modulated in CD4 T cells in response to environmental signals but also that autophagy critically affects the biology of these cells. Mouse models of autophagy deletion in CD4 T cells have indeed shown that autophagy is essential for CD4 T cell survival and homeostasis in peripheral lymphoid organs. Furthermore, autophagy is required for CD4 T cell proliferation and cytokine production in response to T cell receptor activation. Recent developments have uncovered that autophagy controls CD4 T cell differentiation and functions. While autophagy is required for the maintenance of immunosuppressive functions of regulatory T cells, it restrains the differentiation of T_H_9 effector cells, thus limiting their antitumor and pro-inflammatory properties. We will here discuss these findings that collectively suggest that therapeutic strategies targeting autophagy could be exploited for the treatment of cancer and inflammatory diseases.

## Introduction

**Figure 1:**
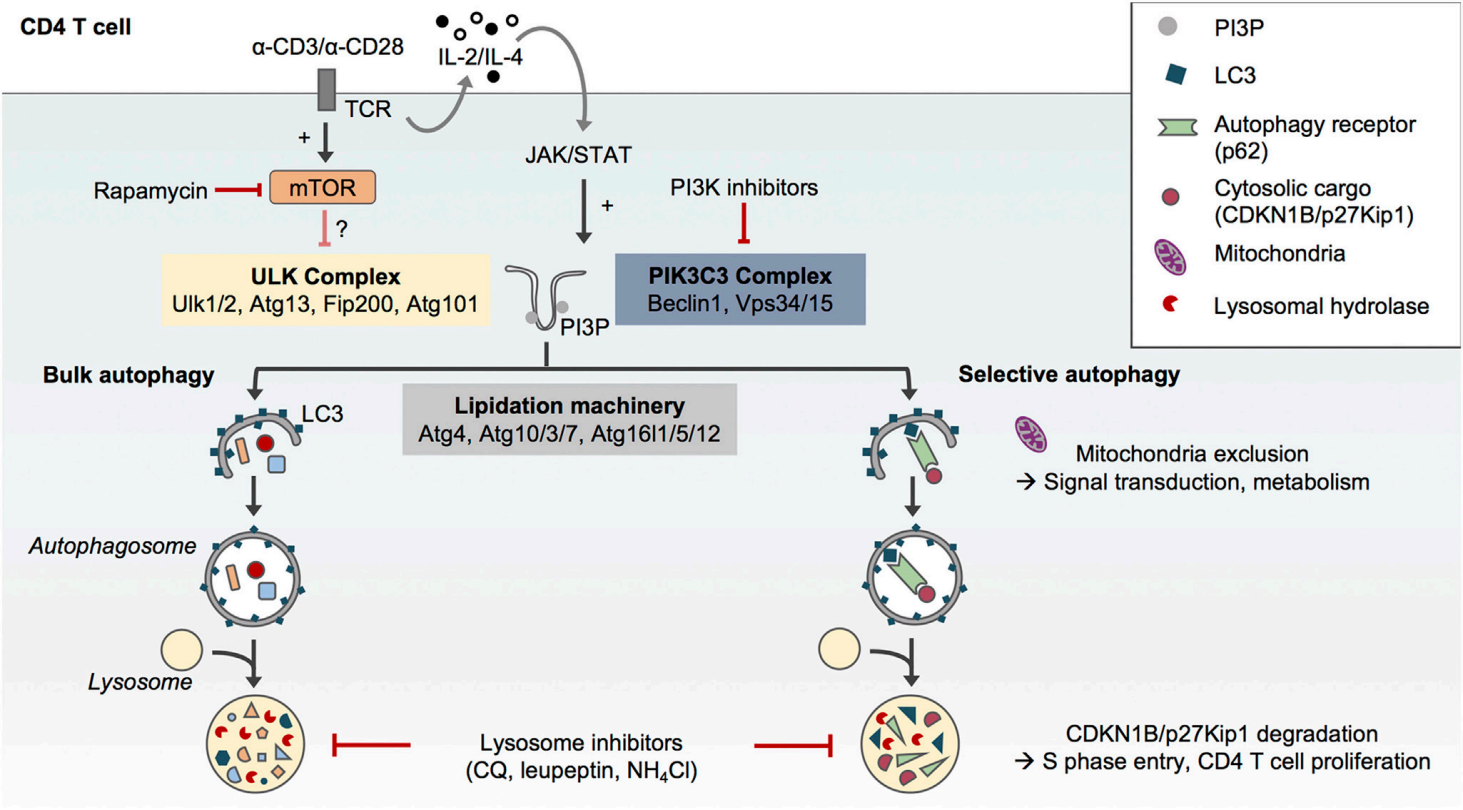
Selective and non-selective autophagy in CD4 T cells upon T cell receptor (TCR) activation. The formation of autophagosomes and autophagic flux in CD4 T cells requires the conventional protein complexes and steps described in other cell types, i.e., the preinitiation complex formed by Ulk1/2, Atg13, Fip200, and Atg101, the phosphatidylinositol-3-kinase (PIK3)C3 complex which comprises Beclin1 and Vps34 and allows for the formation of phosphatidylinositol-3-phosphate (PI3P) and finally the autophagy lipidation machinery which ensures the conjugation of LC3 to phosphatidylethanolamine at the forming autophagosome membrane (1). Autophagy in CD4 T cells can be pharmacologically modulated. While rapamycin may only have a modest impact, PI3K inhibitors such as 3-methyladenine can reduce autophagosome formation in CD4 T cells. Inhibitors of lysosome function such as chloroquine (CQ), leupeptin, or ammonium chloride (NH4Cl) impair autophagic flux. Although TCR engagement activates both mammalian target of rapamycin (mTOR) and autophagy in CD4 T cells, TCR-induced autophagy does not seem to depend on mTOR inhibition but may rather be activated by the JAK/signal transducer and activator of transcription (STAT) pathway in response to autocrine/paracrine interleukin (IL)-2 and IL-4 signaling (4). While CD4 T cells support bulk and selective autophagy (5), the selective degradation of the cell cycle inhibitor CDKN1B/p27Kip1in autophagosomes may contribute to TCR-driven CD4 T cell proliferation (6). Similarly, the exclusion of mitochondria from autophagosome upon TCR activation may contribute to TCR signal transduction and metabolic adaptations (7).

Macroautophagy, hereafter referred to as autophagy, is an evolutionary conserved catabolic pathway that ensures the degradation and recycling of intracellular components. During autophagy, cytosolic proteins and organelles are sequestered in a double membrane-bound structure called the autophagosome and eventually degraded upon fusion of the autophagosome with the lysosome (1). The autophagic flux accordingly refers to a complete catabolic process that ensures the breakdown of cargos and the release of the resulting macromolecules in the cytosol (2). Many autophagy-related (Atg) proteins have been found essential to orchestrate the formation of autophagosomes. This includes the upstream ULK complex (Ulk1/2, Rb1cc1/Fip200, Atg13, and Atg101) which regulates the induction of autophagosome formation. The class III phosphatidylinositol (PtdIns) 3-kinase (PIK3C3/Vps34) complex is then essential for the initial curvature of the phagophore and the recruitment of two ubiquitin-like conjugation systems (Atg4, 3/7/10, and 16l1/5/12) which conjugate Atg8 homologs (microtubule-associated protein 1A/1B-light chain 3/LC3, GABARAPs) to phosphatidylethanolamine and thus ensure the elongation of the phagophore and its closure (Figure 1) (1, 3).

While autophagy was initially described as a nonselective process induced under various stress conditions including nutrient deprivation (8, 9), it is now clear that autophagy can also specifically target organelles, proteins, or pathogens for degradation. This selective autophagy process requires autophagy receptors such as p62/Sqstm1 (5). p62/Sqstm1 recognizes poly-ubiquitinated cargos through its ubiquitin-associated domain and targets them for autophagic degradation through its LC3-interacting region motif (Figure 1). The levels of p62 are thus used as an index of autophagic degradation in combination with LC3 lipidation analysis (2).

Autophagy plays important functions in inflammatory and immune responses (10, 11). Atg proteins are crucial actors of cell-autonomous innate immunity. They contribute to pathogen elimination by antimicrobial selective autophagy (12) and LC3-associated phagocytosis (LAP), a recently identified form of noncanonical autophagy that directs LC3 lipidation onto endolysosomal compartments such as pathogen-containing phagosomes (13, 14). Moreover, both autophagy and LAP indirectly modulate adaptive immunity by contributing to antigen processing and major histocompatibility complex-restricted presentation to T cells (15). Over the last decade, accumulating evidence revealed a cell-intrinsic role for autophagy in CD4 T cell differentiation with direct consequences on physiopathology.

Here, work describing the regulation of autophagy in immune cells will be reviewed, with a focus on the functions of autophagy in CD4 T cell homeostasis and activation. We will also discuss recent findings showing that autophagy modulates the differentiation as well as the effector and regulatory functions of CD4 T cells and how this affects anticancer immune responses.

## Autophagy is Induced in CD4 T Cells upon Activation

The first evidence of autophagosome formation in CD4 T cells was reported 10 years ago by Li and colleagues (16–18). The authors assessed the presence of double membrane-bound autophagosomes in mouse primary CD4 T cells differentiated *in vitro* into effector T helper (T_H_) cells. They focused on T_H_1 and T_H_2 cells, which are, respectively, essential for cell-mediated and humoral immunity (19). Using transmission electron microscopy, they detected autophagosomes in about 20% of T_H_1 and T_H_2 cells activated *in vitro* with anti-CD3 and anti-CD28 antibodies, whereas they did not observe autophagosome in naïve resting CD4 T cells. These findings were confirmed by the expression of exogenous green fluorescent protein (GFP)–LC3 fusion protein in effector T cells and monitoring of GFP–LC3 puncta formation by fluorescence microscopy. With this method, the authors measured the proportion of T_H_1 cells undergoing autophagy in various culture conditions and determined that T cell receptor (TCR) signaling can sustain autophagy in effector CD4 T cells (17). Shortly after, a study conducted by Pua and colleagues gave further support to these data by detecting increased levels of LC3 lipidation by Western blot in primary mouse CD4 T cells cultured in the presence of anti-CD3 antibodies (18). Accordingly, both reports showed for the first time that key autophagy genes Atg5, Atg7, Beclin1, and LC3 are expressed in CD4 T cells (17, 18). They also found that downregulation of the expression of these genes and inhibition of Jun amino-terminal kinase (JNK)/mitogen-activated protein kinase pathways or PtdIns-3-kinase (PI3K) could inhibit autophagy in CD4 T cells, whereas the inhibition of mammalian target of rapamycin (mTOR) led to autophagy induction (17). These two initial reports, which evidenced that autophagy is induced in CD4 T cells upon TCR activation, were confirmed by several later studies conducted in mouse (4, 7, 20–22) and human primary CD4 T cells (23). In line with these studies, the expression of some autophagy proteins increases upon TCR activation. The activation of primary mouse CD4 T cells results in increased Beclin1 protein levels possibly after the activation of Becn1 promoter by p65/NF-κB (24). Upregulation of LC3 protein levels upon the activation of naïve CD4 T cells and the reactivation of differentiated effector CD4 T cells has also been reported. Collectively, these studies indicate that the molecular mechanisms of autophagy in CD4 T cells are similar to those described in other cell types and that this pathway can be modulated by pharmacological and genetic approaches.

## Molecular Mechanisms Regulating Autophagosome Formation in CD4 T Cells

While TCR activation activates autophagosome formation in CD4 T cells, it has also been shown to induce mTOR activation (25). Botbol and colleagues have interrogated the involvement of mTOR in TCR-induced autophagy. To measure autophagic flux, the authors monitored LC3 lipidation in effector T_H_1 and T_H_2 cells cultured in various conditions in the presence of the inhibitors of lysosome function ammonium chloride (NH_4_Cl) and leupeptin. Surprisingly, effector T_H_1 and T_H_2 CD4 T cells reactivated with anti-CD3 and anti-CD28 antibodies did not display an increased autophagic flux upon mTOR inhibition with rapamycin, suggesting that this process is mTOR-independent. However, it cannot be excluded that T_H_1 and T_H_2 CD4 T cell reactivation on its own increased autophagic flux to its maximal level. This result may also suggest that TCR-induced autophagy signaling pathways other than mTOR can be involved in the regulation of autophagy in CD4 T cells such as the Janus tyrosine kinase (JAK)/signal transducer and activator of transcription (STAT) signaling pathway. Indeed, the γ-chain cytokines interleukin (IL)-2 and IL-4, which are, respectively, produced by T_H_1 and T_H_2 cells upon reactivation, have been shown to contribute to autophagy induction in effector CD4 T cells in an autocrine/paracrine and JAK3-dependent manner (Figure 1) (4).

Data from the literature collectively suggest that autophagosome formation in CD4 T cells requires the canonical steps and molecules previously described in other cell types. For instance, overexpression of a kinase-dead mutant of the upstream autophagy protein ULK1 (ULK1 K461) in human naïve CD4 T cells impairs LC3 lipidation and autophagy (23). Likewise, reduced levels of autophagy have been described in CD4 T cells lacking the PI3K complex component Beclin1 (26) or the autophagy lipidation machinery proteins Atg7 (17, 18, 27), Atg3 (20), and Atg5 (28) (Figure 1). The requirement of PtdIns-3-phosphate (PI3P) formation by Vps34 during autophagy in CD4 T cells remains, however, elusive. Conditional deletion of Vps34 in mouse CD4 T cells does not completely suppress LC3 lipidation, and the PI3K inhibitor 3-methyladenine inhibits LC3 lipidation in Vps34-deficient cells, underscoring a possible contribution of Vps34-independent additional sources of PI3P in these cells (29–31). These findings can also indicate that noncanonical autophagy leading to endolysosomal LC3 lipidation may be active in CD4 T cells, a question that remains to be addressed.

Over the last decade, studies conducted in various models of autophagy deletion allowed for a better understanding of the molecular signals which control autophagy in CD4 T cells. These studies and others also strongly contributed to the understanding of the functions of autophagy in CD4 T cells. Indeed, while the early studies, which elegantly uncovered autophagosome formation in CD4 T cells, also suggested that autophagy could contribute to CD4 T cell death *in vitro* in response to specific stimuli (16, 17), they did not clearly ascribe a physiological function to autophagy in these cells.

## Autophagy Maintains CD4 T Cell Homeostasis

The first study aiming at exploring the role of autophagy in CD4 T cell biology was conducted by Pua and colleagues in 2007. The authors generated autophagy-deficient T cells by transferring Atg5-deficient fetal hematopoietic progenitors into lethally irradiated wild-type congenic hosts and investigated how autophagy contributes to T cell development and functions. The chimeric mice generated by Pua and colleagues displayed a reduced thymus cellularity as well as severely altered CD4 and CD8 T cell compartments in peripheral lymphoid organs. The authors attributed these results to the defective capacity of Atg5^−/−^ lymphoid progenitors to reconstitute lymphoid compartments and the impaired survival of Atg5^−/−^ CD8 and, to a lesser extent, CD4 T cells in the periphery (18). Since Atg5 was known to interact with proteins from the apoptosis pathway (32), Pua and colleagues generated a second autophagy-deficient mouse model. To overcome the possible effect of autophagy deletion on hematopoietic progenitor cell proliferation capacity, they used an Atg7^fl/fl^:Lck-Cre system which allows for the deletion of Atg7 from the double-positive stage of thymocyte development. Analyses of thymocytes and mature T cells revealed a decrease in CD4 and CD8 single-positive thymocyte numbers and a dramatic loss of CD4 and CD8 T cells in the peripheral lymphoid organs of Atg7^fl/fl^:Lck-Cre animals, indicating a critical role for autophagy in maintaining peripheral CD4 T cell homeostasis (33). Along with lymphopenia in peripheral lymphoid organs, Pua and colleagues noted the acquisition of a “memory-like phenotype” by CD4 T cells characterized by a reduction in CD62L membrane levels and an increase in CD44 membrane levels (CD44^high^/CD62L^low^) (33). These findings, which were further supported by several studies conducted in other models of autophagy deletion in mouse T cells, also demonstrated that autophagy is required for peripheral CD4 T cell survival (Table 1) (7, 20, 28–31, 34–36). Indeed, CD4 T cells isolated from Atg7^fl/fl^:Lck-Cre mice displayed increased apoptosis levels as shown by Annexin V and active caspase staining together with an imbalance of anti- and pro-apoptotic proteins. Consistent with the role of autophagy in eliminating damaged organelles and protecting from cell death, Atg7-deficient CD4 T cells displayed an increased mitochondrial content and levels of reactive oxygen species (ROS) as well as an impaired regulation of mitochondrial number during their development (33). Supporting this idea, transcriptomic analyses conducted in Atg5-deficient thymocytes revealed an enrichment of transcripts encoding mitochondrion-associated proteins which can account for the increased mitochondrial mass observed in peripheral mature autophagy-deficient CD4 T cells (28). Furthermore, *in vitro* deletion of Atg3 in splenic T cells from Atg3^fl/fl^ estrogen receptor-Cre mice had no acute effect on organelle homeostasis and CD4 T cell survival but induced temporal accumulation of mitochondria and endoplasmic reticulum cell death after long-term culture. This suggested that the effect of autophagy deletion on peripheral CD4 T cell homeostasis is due to an accumulation of defects during their development rather than an acute phenomenon (20).

**Table 1:** Autophagy and CD4 T cell homeostasis.

	Mouse model of autophagy deletion	Phenotypic observations					
		Reduced SP thymocyte numbers	Reduced peripheral CD4 T cell numbers	Memory-like phenotype of CD4 T cells	Increased CD4 T cell apoptosis	Increased mitochondrial content	Other observations
Pua et al. (18)	Atg5^−/−^ chimeras	X	X		X		
Pua et al. (33)	Atg7^fl/fl^:Lck-Cre	X	X	X	X	X	
Stephenson et al. (28)	Atg5^fl/fl^:Lck-Cre Atg7^fl/fl^:Lck-Cre	X	X	X	X	X	
Hubbard et al. (7)	Atg7^fl/fl^:Lck-Cre Atg7^fl/fl^:ER-Cre	X	X				
Jia and He (20)	Atg3^fl/fl^:Lck-Cre	X	X	X	X		
Mcleod et al. (29)	Vps34^fl/fl^:Lck-Cre	X	X	X	X		
Kovacs et al. (34)	Becn1^fl/fl^:CD4-Cre		X				
Willinger and Flavell (30)	Vps34^fl/fl^:CD4-Cre		X			X	Increased CD8 T cell apoptosis
Parekh et al. (31)	Vps34^fl/fl^:CD4-Cre		X	X	X	X	
Puleston et al. (35)	Atg7^fl/fl^:CD4-Cre Atg5^fl/fl^:vav-Cre		X	X (in CD8 T cells)			Impaired memory CD8 T cell formation
Kabat et al. (36)	Atg1611^fl/fl^:CD4-Cre		X	X			Reduced peripheral Foxp3 Treg cell numbers

*Phenotypic observations made from different mouse models of autophagy deficiency reported in the literature and identifying a role for autophagy in CD4 T cell homeostasis*.

*SP, single positive; Lck, lymphocyte protein tyrosine kinase; ER, estrogen receptor; Cre, Cre recombinase; vav, vav guanine nucleotide exchange factor 1*.

While further studies based on the deletion of Vps34 in CD4 T cells confirmed the critical role of autophagy in maintaining CD4 T cell survival during their development (30, 31), it was proposed that autophagy and autophagy proteins could favor CD4 T cell survival upon TCR stimulation. While Pua and colleagues initially suggested that *in vitro* TCR activation with anti-CD3 antibodies leads to the apoptotic cell death of Atg5-deficient CD4 T cells isolated from Atg5^−/−^ chimeric mouse splenocytes (18), further work conducted in mouse models of Atg7 deletion found similar levels of apoptosis in Atg7-competent and -deficient CD4 T cells upon *in vitro* TCR stimulation (6, 7). Conversely, targeting upstream components of the autophagy pathway seems to impair CD4 T cell survival upon TCR stimulation. Indeed, Kovacs and colleagues showed that the deletion of Beclin1 in CD4 T cells led to increased apoptosis upon TCR activation with anti-CD3 and anti-CD28 antibodies as indicated by increased levels of the pro-apoptotic genes Bim and pro-caspases 8 and 3 as well as DNA fragmentation. The authors proposed that the degradation of pro-caspase 8 by autophagy prevents its accumulation in protein complexes that function as signaling platforms to activate apoptosis. The ability of caspase inhibitor addition and modulation of pro- and antiapoptotic protein expression levels to prevent cell death further supported the apoptosis-mediated death of autophagy-deficient cells (34). Similarly, overexpression of a kinase-dead mutant of ULK1 in human CD4 T cells has been shown to induce mitochondria and ROS accumulation leading to CD4 T cell apoptosis upon TCR stimulation (23). It is worth noting that autophagy-independent functions Vps34 have also been shown to modulate CD4 T cell survival. Indeed, Vps34 deletion leads to CD4 T cell death, independently of autophagy and rather through impairment of trafficking and surface expression of IL-7 receptor, and regulation of IL-7 signaling (29).

By controlling activation-induced death of CD4 T cells but also their proliferation, differentiation, and cytokine production, TCR engagement is crucial for CD4 T cell homeostasis. Early after the first observation of autophagosomes in CD4 T cells, autophagy has not only been shown to be activated upon TCR engagement but also to modulate CD4 T cell responses to this signal.

## Autophagy Controls CD4 T Cell Proliferation in Response to TCR Activation

Along with demonstrating the crucial role of autophagy for CD4 T cell homeostasis and survival in peripheral organs, Pua and colleagues also showed that autophagy induction following TCR activation promotes TCR-driven proliferation of CD4 T cells. Analysis of cell proliferation by 5(6)-carboxyfluorescein N-hydroxysuccinimidyl ester dilution revealed that Atg5-deficient CD4 T cells display impaired proliferation following *in vitro* TCR activation with anti-CD3 antibodies, anti-CD28 antibodies, and IL-2. However, the normal levels of membrane TCR and activation markers CD69 and CD25 indicated that autophagy inhibition does not alter TCR-driven activation of CD4 T cells (18). While these findings were confirmed later in other mouse models of Atg3, Atg5, Atg7, and Vps34 deletion in CD4 T cells (6, 7, 20, 28, 31), several groups aimed at uncovering the molecular mechanisms linking autophagy and CD4 T cell responses to TCR activation.

Jia and colleagues proposed that the selective degradation of the cell cycle inhibitor CDKN1B/p27Kip1 by p62-dependent autophagy may account for the TCR-driven proliferation defect observed in autophagy-deficient cells, as Atg3- and Atg7-deficient CD4 T cells accumulate CDKN1B/p27Kip1 and fail to enter S phase after *in vitro* TCR stimulation (Figure 1) (6). In an earlier study, the same group demonstrated that Atg7 deletion in CD4 T cells led to the accumulation of endoplasmic reticulum and impaired calcium mobilization upon *in vitro* TCR stimulation associated with an increased endoplasmic reticulum stress (37). However, this calcium mobilization defect does not seem to affect CD4 T cell activation since Atg7-deficient CD4 T cells display intact proximal TCR signaling and NF-κB pathway and maintain IL-2 production upon *in vitro* TCR activation (37).

The induction of autophagy upon TCR activation has also been proposed to regulate the energy metabolism changes required for CD4 T cell activation. Indeed, in T_H_1 cells, both pharmacological and genetic inhibition of autophagy impair the production of ATP, interferon-gamma (IFN-γ), and IL-2 following TCR activation. These defects of autophagy-deficient T_H_1 cell functions can be reversed by the addition of methyl pyruvate, a cell-permeable glucose metabolism intermediate that can restore electron transport chain, and oxidative phosphorylation activity. Interestingly, the analysis of autophagosome content in T_H_1 cells revealed a change in autophagic cargos from mitochondria to soluble cytosolic component upon TCR activation (Figure 1). The authors proposed that this selective exclusion of mitochondria from autophagic degradation in response to TCR engagement may contribute to signal transduction and adaptation to energy requirement modification (7). Mitochondrion remodeling has been shown to control T cell metabolic adaptation, driving their response and fate following TCR activation, reinforcing the central role of this organelle in CD4 T cell biology (38).

A contrasting study conducted in effector T_H_2 cells has proposed that selective autophagy may prevent a sustained TCR activation by targeting the adaptor protein B-cell CLL/lymphoma 10 for degradation and thus limiting TCR-driven NF-κB activation. Nevertheless, this process does not seem to contribute to naive CD4 T cell response to TCR activation and has not been tested in other effector CD4 T cell subsets, suggesting that it may be limited to T_H_2 cells (39).

Collectively, these data demonstrated the importance of autophagy for CD4 T cell homeostasis not only during their development but also upon activation. These results are in line with increased levels of autophagy detected in the CD4 T cells of rheumatoid arthritis that may account for their hyperactivation and resistance to apoptosis (22). Until now, the specific role of autophagy in the different subtypes of effector and regulatory T cells has received less attention. Recent studies have shown that the function of autophagy may vary according to the subsets of CD4 T cells and most importantly that autophagy modulates CD4 T cell differentiation and functions by regulating energy metabolism or intracellular component levels.

## Autophagy Regulates Effector and Regulatory CD4 T Cell Differentiation and Functions

**Figure 2:**
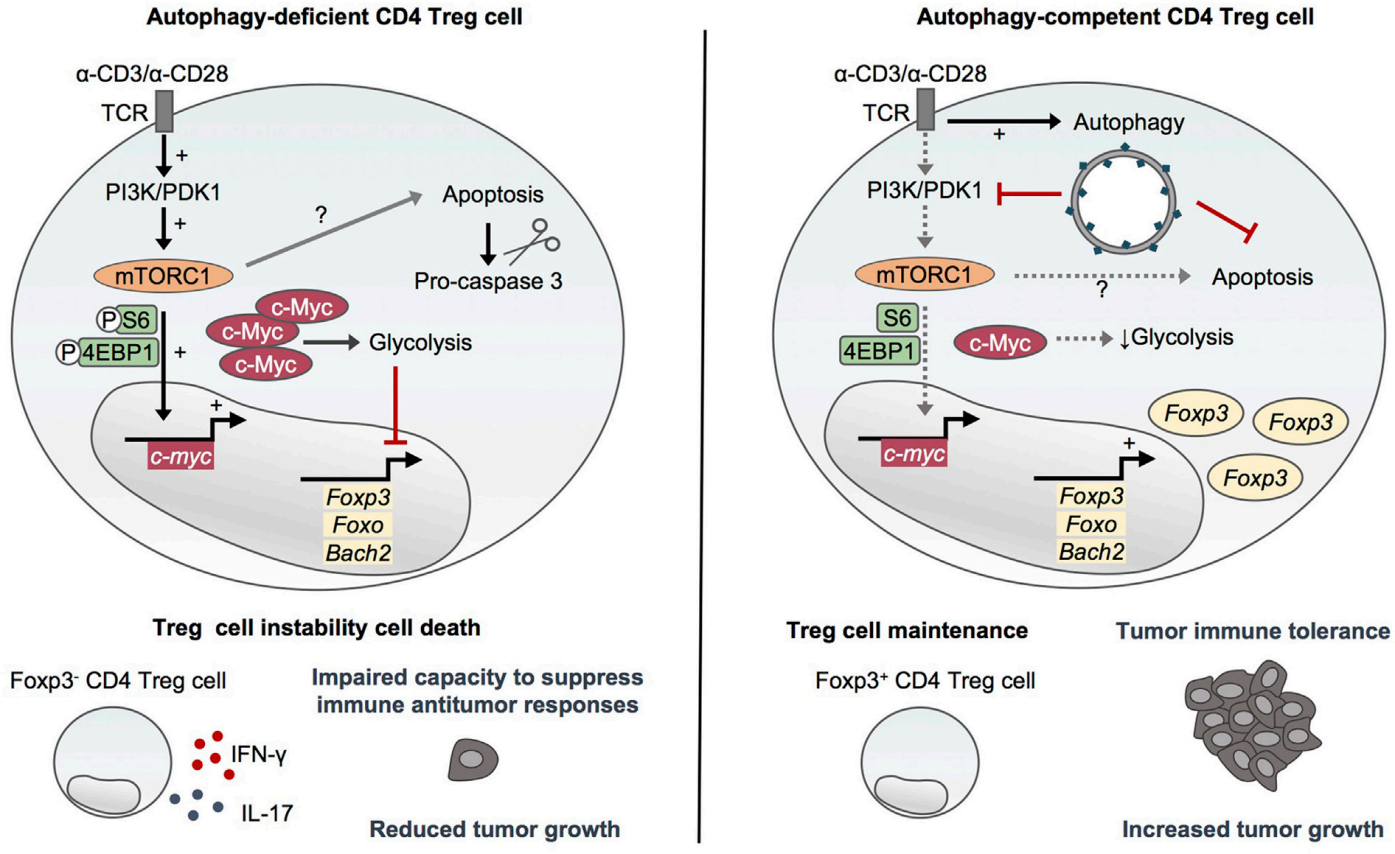
Autophagy maintains CD4 Treg cell stability and immunosuppressive functions. Activating environmental signals are essential for the functional maturation of CD4 Treg cells and activate both mammalian target of rapamycin (mTOR)C1 and autophagy. Autophagy-deficient CD4 Treg cells lacking Atg5 or Atg7 display an increased mTORC1 activity and upregulation of c-Myc and glycolytic metabolism (36, 40). This dysregulation of mTORC1/c-Myc pathway and energy metabolism leads to the loss of Foxp3, Foxo, and Bach2 which are essential for CD4 Treg cell differentiation and maintenance, and to an aberrant production of inflammatory cytokines (40). Furthermore, autophagy deficiency impairs CD4 Treg cell survival through a mechanism that seems to only partly rely on mTORC1 activity (36, 40). Autophagy thus plays a crucial role in maintaining the stability and functional integrity of CD4 Treg cells by restraining mTORC1–c-Myc pathway and glycolytic metabolism as well as promoting survival. Importantly, autophagy is required to maintain the ability of CD4 Treg cells to suppress antitumor immune responses *in vivo* (40).

In 2016, Wei and colleagues reported that autophagy is essential to maintain the functional integrity of the suppressor CD4 Foxp3^+^ Treg cells (40), which suppress effector T cell functions (41, 42). The specific deletion of Atg7 in Foxp3^+^ CD4 T cells leads to reduced proportions of Foxp3^+^ CD4 T cells in peripheral organs and a higher active caspase-3 staining of the remaining cells, indicating a survival defect of autophagy-deficient Treg cells (40). The authors reported an increased proportion of Ki67-positive CD4 Treg cells purified from Atg7^fl/fl^:Foxp3Cre mice, probably reflecting a niche-filling behavior of the surviving cells. However, Atg7-deficient Treg cells proliferate normally in response to *in vitro* TCR stimulation with anti-CD3 antibodies, anti-CD28 antibodies, and IL-2 and after adoptive transfer into Rag1^−/−^ mice. This suggests that Atg7 may be dispensable for TCR-induced proliferation of Treg cells (40). Compared to autophagy-competent cells, Atg7-deficient Treg cells display an increased glycolytic activity upon TCR activation. This reveals an important role for autophagy in negatively regulating glucose metabolism in Treg cells by restraining the mTOR complex 1 (mTORC1)–c-Myc pathway. Indeed, the high mTORC1 activity observed in activated Atg7-deficient Treg cells and characterized by high phosphorylation levels of ribosomal protein S6 (S6) and eukaryotic translation factor 4E binding protein 1 (4EBP1) has been shown to rely on increased PI3K and pyruvate dehydrogenase kinase 1 abundance and activation and to be responsible for upregulated c-Myc expression and altered transcription programs (40). Importantly, autophagy controls transcriptional programs in Treg cells in a similar mTORC1-dependent manner. Atg7-deficient Treg cells display a reduced expression of Foxp3, Foxo, and Bach2, as well as an enrichment of effector T cell differentiation pathways that can be rescued by mTORC1 inhibition with rapamycin. This indicates a central role for autophagy in negatively regulating effector programs and maintaining Treg cell stability. These results are in line with the loss of Foxp3 expression and the aberrant production of IFN-γ and IL-17 observed in Atg7-deficient Treg cells *in vitro* and *in vivo* (Figure 2). Moreover, autophagy is required for the ability of Treg cells to suppress antitumor responses *in vivo* as illustrated by an impaired tumor growth of MC38 colon adenocarcinoma cells in Atg7^fl/fl^:Foxp3Cre mice associated with a loss of Treg cells and an increased expression of IFN-γ from T cells at the tumor site. Although Atg7 deficiency also leads to survival defects of Treg cells, it is worth noting that autophagy seems to repress Treg cell apoptosis through a mechanism that does not solely rely on mTORC1 and which may thus be distinct from its role in Treg cell stability and functional integrity (40) (Figure 2).

In line with this, Le Texier and colleagues reported that the specific deletion of Atg7 in Foxp3 + CD4 T cells leads to the profound loss of the Helios + TIGIT + subset of Treg cells in the spleen and the bone marrow. The authors showed that autophagy is required for the survival of these Helios + TIGIT + Foxp3 + CD4 T cells and the maintenance of their immunosuppressive functions. Indeed, aged Atg7^fl/fl^:Foxp3Cre mice develop spontaneous T cell activation and multi-organ inflammation, suggesting an important role for autophagy-dependent Treg cells in the suppression of autoimmunity. Importantly, Treg-intrinsic autophagy promotes Treg reconstitution upon stem cell transplantation and attenuates graft versus host disease, supporting the idea that autophagy-dependent Treg cells are critical for tolerance (27).

In another model, the specific deletion of Atg16l1 in both CD4 T cells and Foxp3^+^ CD4 T cells results in a loss of Treg cells in mouse intestine and aberrant expression of the effector cytokines IFN-γ and IL-17 by the remaining Treg cells, associated with T_H_2-driven intestinal inflammation toward dietary antigens and commensal microbiota (36). In this study, no evidence of defective *in vitro* Treg cell differentiation or stability was detected. Thus, Kabat and colleagues attributed the impairment of Atg16l1-deficient Treg cell survival to their aberrantly high glycolytic metabolism which prevents their metabolic adaptation to the intestinal mucosal environment. Indeed, the authors found that glycolysis gene expression levels are higher in Atg16l1-deficient Treg cells compared to their autophagy-competent counterparts, especially in Foxp3^+^ Treg cells sorted from the colon lamina propria of young Atg16l1-deficient mice. Furthermore, Treg cells differentiated *in vitro* from Atg16l1-deficient naive CD4 T cells display higher expression levels of c-Myc and a panel of glycolysis genes, reduced expression levels of lipid metabolism-associated genes as well as higher rates of glycolysis and oxidative phosphorylation than autophagy-competent Treg cells, confirming that autophagy negatively controls glucose metabolism in Treg cells (36).

This study also revealed that autophagy deletion has an opposite effect on T_H_2 cells, promoting their expansion in peripheral tissues through both Treg cell-mediated control and cell-intrinsic mechanism. Glucose metabolism is not altered by autophagy deficiency in effector T_H_2 cells which display high levels of c-Myc and glycolysis gene expression as well as glycolytic energy metabolism, irrespective of their Atg16l1 genotype (36), consistent with their high glycolytic rate compared to other CD4 T cell subsets (43). The constitutively high levels of glycolysis displayed by T_H_2 cells may allow them to adapt to the metabolic switch toward glycolysis induced by autophagy deficiency. GATA-3 having been previously associated with c-Myc-driven metabolic reprogramming leading to glycolysis induction upon TCR activation (44), the authors proposed that this transcription factor essential to T_H_2 cell differentiation may orchestrate the metabolic adaptations induced by autophagy deficiency. This suggests that the differential expression of transcription factors such as GATA-3 in the various subsets of regulatory and effector CD4 T cells may account for the different metabolic adaptations to autophagy deficiency (36). Together, these studies emphasize how the contribution of autophagy in energy metabolism varies according to the subsets of CD4 T cells, explaining, at least partly, the differential impact of autophagy deletion on CD4 T cell subset differentiation, stability, and functions.

In line with this, our recent study focused on the role of autophagy in the differentiation and functions of T_H_9 cells, characterized as IL-9 producing effector T cells which contribute to autoimmune diseases (45, 46) and exert potent anticancer functions (47–51). Using pharmacological and genetic approaches of autophagy inhibition in CD4 T cells, we showed that autophagy restrains T_H_9 cell differentiation and effector functions through a cell-intrinsic mechanism independent of energy metabolism modulation (52, 53). While we confirmed that mTORC1 activity is enhanced in Atg5-deficient *in vitro* differentiated Treg cells compared to autophagy-competent controls, we observed no difference in S6 and 4EBP1 phosphorylation in Atg5^−/−^ CD4 T cells differentiated *in vitro* in effector T_H_9 cells, indicating that autophagy does not control mTORC1 signaling and thus glycolytic metabolism in T_H_9 cells. We proposed that T_H_9 cells display similar responses than T_H_2 cells regarding glycolytic metabolism in the absence of autophagy (53).

**Figure 3:**
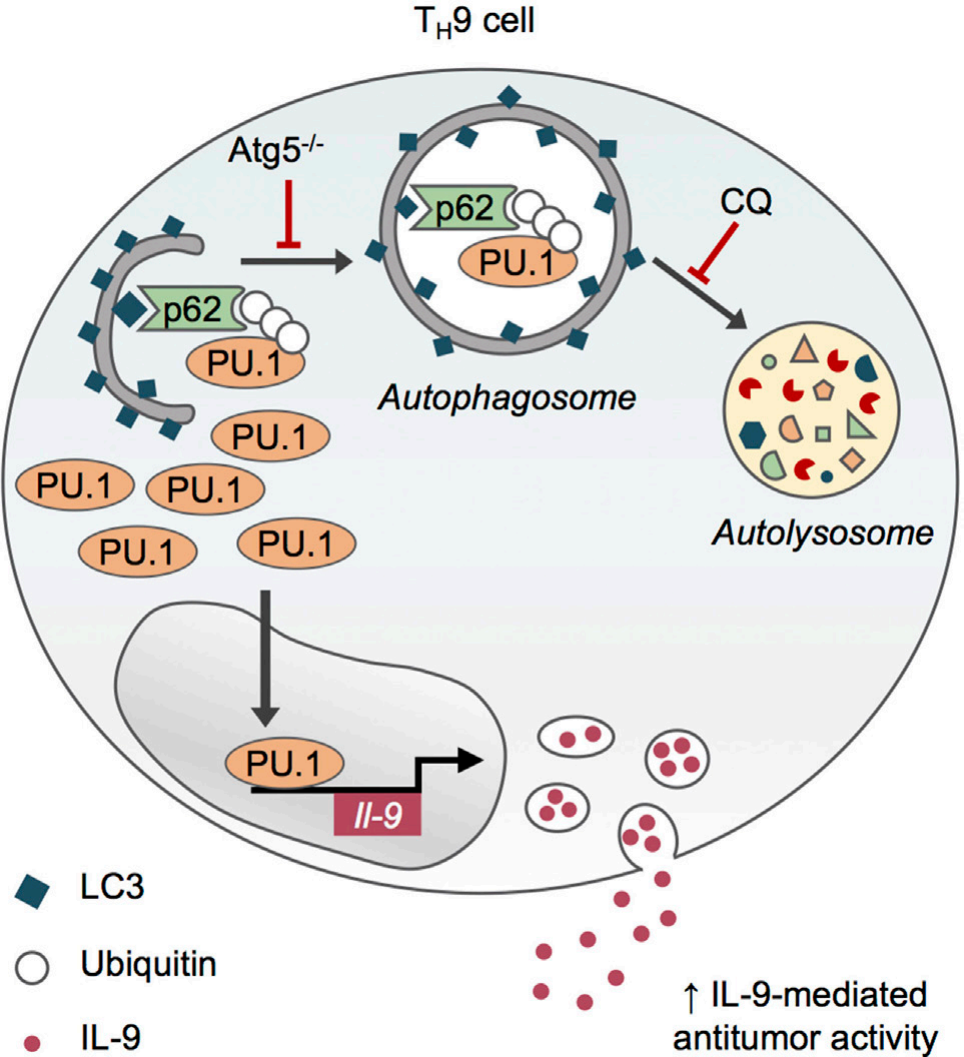
Selective autophagy limits T_H_9 cell differentiation and antitumor functions. Upon TH9 cell differentiation, K63 ubiquitination of PU.1, the master transcription factor of TH9 cells, leads to its specific recruitment by the autophagy receptor p62 *via* its ubiquitin-associated domain and the subsequent degradation of PU.1 in autophagosomes. Genetic (Atg5^−/−^) and pharmacological inhibition of autophagy with chloroquine (CQ) prevent PU.1 degradation and enhance IL-9 secretion from TH9 cells, thus increasing their antitumor functions *in vivo* (53).

Although the inhibition of autophagy through genetic (Atg5 deletion in CD4 T cells) and pharmacological chloroquine approaches reduces the viability of *in vitro* differentiated effector and regulatory CD4 T cells, it specifically enhances IL-9 production and T_H_9 cell differentiation *in vitro*, without affecting the differentiation of other effector T cell subsets or skewing them toward T_H_9 differentiation program (52, 53). This role for autophagy in repressing IL-9 production by differentiating T_H_9 cell relies on the degradation of PU.1, the master T_H_9 cell transcription factor (54), by selective autophagy. Indeed, upon T_H_9 cell differentiation, K63 ubiquitination of PU.1 leads to its specific recruitment by the autophagy receptor p62 *via* its ubiquitin-associated domain and the subsequent degradation of PU.1 in autophagosomes (Figure 3). T_H_9 cells treated with the inhibitor of lysosome function chloroquine (CQ) display increased IL-9 secretion and antitumor properties upon adoptive transfer. Indeed, CQ-treated *in vitro* differentiated T_H_9 cells have enhanced suppressive activity against melanoma tumor growth compared to T_H_9 cells displaying intact autophagic flux, suggesting that CQ may be considered in the context of adoptive T cell therapy of cancer (52, 53). Furthermore, genetic inhibition of autophagy in T cells leads to IL-9-dependent inhibition of tumor growth in mice bearing MC38 colon cancer and B16 melanoma and enhanced IL-9-producing CD4 tumor-infiltrating lymphocytes. While these results lend further support to the potent anticancer functions of T_H_9 cells (Figure 3), they suggest that autophagy induction in tumor microenvironment represses T_H_9 cell development and/or function, potentially explaining the low frequencies of T_H_9 cells detected in tumor lesions (48). This also suggests that inhibiting autophagy may restore antitumor immunity by concomitantly promoting T_H_9 cell-dependent antitumor immunity and impairing Treg cell stability (40), providing new opportunities for cancer immunotherapy.

Through their pro-inflammatory functions, T_H_9 cells play an important role in the development of inflammatory bowel diseases (IBDs). Indeed, IL-9-secreting T_H_9 cells induce colitis in mice (45, 55), and PU.1-expressing cells as well as target cells expressing IL-9 receptor are frequently detected in the gut mucosa of IBD patients (56, 57). Autophagy contributes to inflammation, and IBD pathogenesis through multiple processes (58, 59) and mutations in Atg genes such as *NOD2, ATG16L1*, and *ULK1* leading to autophagy deficiency have been associated with IBD susceptibility in humans (59). Our findings raise the hypothesis that autophagy may limit inflammation by repressing T_H_9 cells’ inflammatory properties. Combined with the evidence that autophagy plays a central role in Treg cell-mediated immune tolerance in peripheral tissues (36, 40, 60) and limits T_H_2 expansion in intestinal mucosa, they provide new insights that could be exploited for therapies against allergies as well as inflammatory and autoimmune diseases.

Collectively, investigators have demonstrated that autophagy levels in CD4 T cells are regulated in response to environmental signals and that autophagy controls CD4 T cell homeostasis and functions. The molecular mechanisms, which contribute to autophagy-driven modulation of effector and regulatory T cell functions, have also been clarified. The role for autophagy inhibition in restraining Treg cell functions and promoting T_H_9 cell differentiation evidenced in recent studies opens new therapeutic perspectives regarding the combination of autophagy inhibitors with anticancer immunotherapies. Recent cancer clinical trials have suggested that the clinically approved autophagy inhibitor hydroxychloroquine may be considered for cancer therapy although there is still a need for the development of more specific and potent autophagy inhibitors (61). Further work is now required to determine the effect of such treatments on CD4 T cell autophagy, differentiation, and functional integrity *in vivo*.

## Author Contributions

EJ and LA wrote the manuscript and EJ designed the figures.

## Conflict of Interest Statement

The authors declare that the research was conducted in the absence of any commercial or financial relationships that could be construed as a potential conflict of interest.
